# Colonoscopy‐assisted percutaneous sigmoidopexy for a complete rectal prolapse: A case report

**DOI:** 10.1002/deo2.175

**Published:** 2022-10-17

**Authors:** Junji Takahashi, Masashi Yoshida, Teppei Kamada, Yuichi Nakaseko, Keigo Nakashima, Norihiko Suzuki, Hironori Ohdaira, Yutaka Suzuki

**Affiliations:** ^1^ Department of Surgery International University of Health and Welfare Tochigi Japan

**Keywords:** abdominal wall, colonoscopy, endoscopy, fecal incontinence, rectal prolapse

## Abstract

Colonoscopy‐assisted percutaneous sigmoidopexy is a simple and swift procedure that does not require general anesthesia. While we first developed this procedure for treating sigmoid volvulus, we herein present the first case in which we used it to correct a complete rectal prolapse in an older patient. Existing treatment modalities for rectal prolapses are limited by high recurrence rates, greater invasiveness, and greater complications; thus, there is a need for minimally invasive techniques that are associated with lower recurrence rates and fewer complications. In this case, a woman in her 90s complained of persistent fecal incontinence, dysuria, anal pain, and difficulty in walking. She was diagnosed with a complete rectal prolapse of 15 cm and was treated with colonoscopy‐assisted percutaneous sigmoidopexy. The sigmoid colon was tractioned colonoscopically and fixed to the abdominal wall to immobilize the prolapsed rectum. The patient developed no complications intraoperatively and postoperatively and experienced no recurrence during a 5‐year postoperative period. This report documents the first case wherein colonoscopy‐assisted percutaneous sigmoidopexy was used successfully to correct a complete rectal prolapse.

## INTRODUCTION

We originally developed colonoscopy‐assisted percutaneous sigmoidopexy (CAPS) as a technique to eliminate the need for a colon resection or stoma installation during the treatment of sigmoid volvulus and achieved very good results regarding the fixation of an elongated bowel.^1^ During CAPS, a twisted sigmoid colon is reverted to its normal position and fixed to the abdominal wall. We have previously performed CAPS in eight cases without any complications or major recurrences.^1^ We speculated that the CAPS concept of fixating the intussuscepted intestinal tract to the anterior abdominal wall could achieve similar results when applied to cases of rectal prolapse. As far as we know, there are no existing reports on utilizing CAPS for correcting a complete rectal prolapse.

A complete rectal prolapse is a circumferential prolapse of the rectum from the anus; it occurs more commonly in older women^2^, ^3^, ^4^ (median age: 80 years) and is associated with fecal incontinence. Usually, a surgical approach is required to correct the condition; however, even general anesthesia is risky in many of these patients due to their comorbidities. Thus, this susceptible and vulnerable population requires a simple, safe, and reliable surgical method that can correct a complete rectal prolapse without relying on general anesthesia or colon resection.

We present a case of a complete rectal prolapse that was treated by CAPS, which is one such simple technique that requires only endoscopy‐guided sigmoidopexy without general anesthesia or colon resection.

## CASE REPORT

A woman in her 90s complained of persistent fecal incontinence, dysuria, anal pain, and difficulty in walking. The patient expressed suicidal thoughts because these symptoms had significantly impacted her quality of life.

**FIGURE 1:**
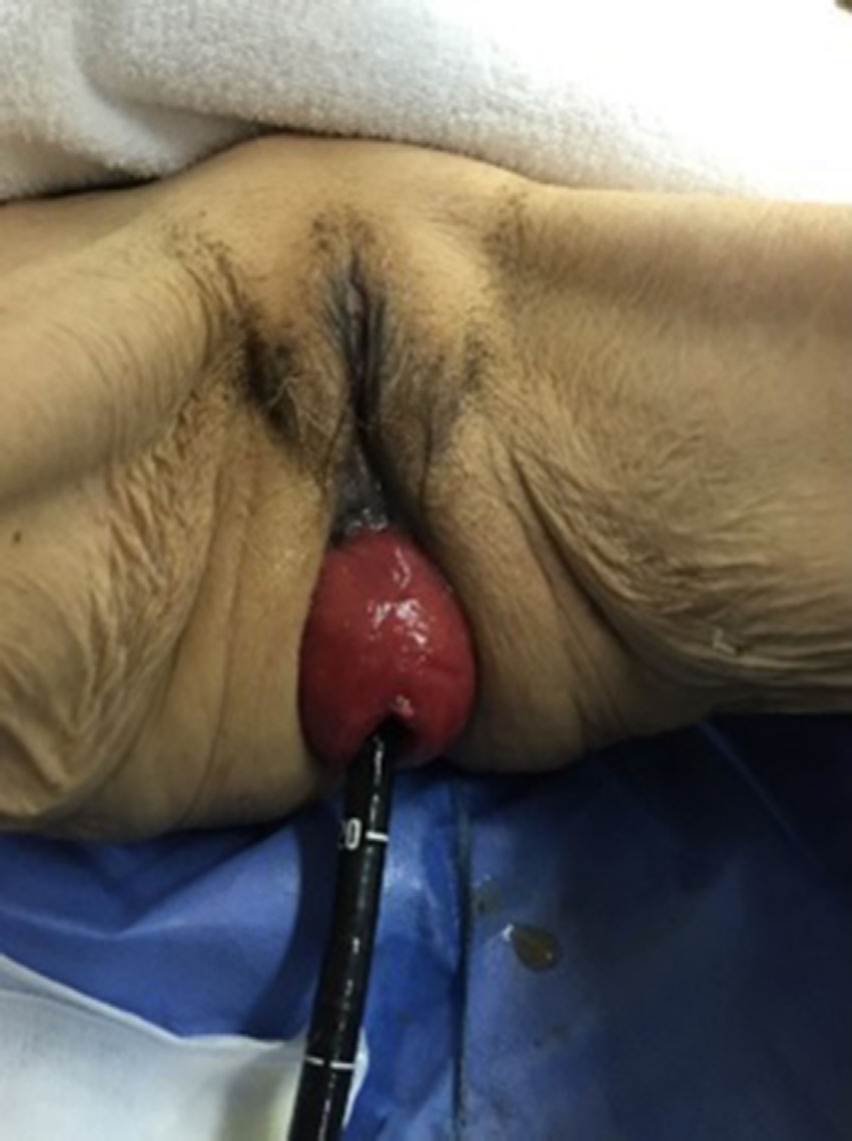
The rectum is prolapsed 15 cm from the anus in a circumferential fashion

Physical examination revealed a complete rectal prolapse of 15 cm (Figure 1); the patient was unable to ambulate due to this condition. The Barthel index, a measure of mobility and performance in activities of daily living, was 5. The American Society of Anesthesiologists Physical Status Classification system score, a measure of physical status and perioperative risks, was 4. The patient had a severe systemic disease in the form of cardiac diastolic dysfunction and tricuspid regurgitation, which made invasive operative procedures under general anesthesia risky.

### Sigmoidopexy technique

**FIGURE 2:**
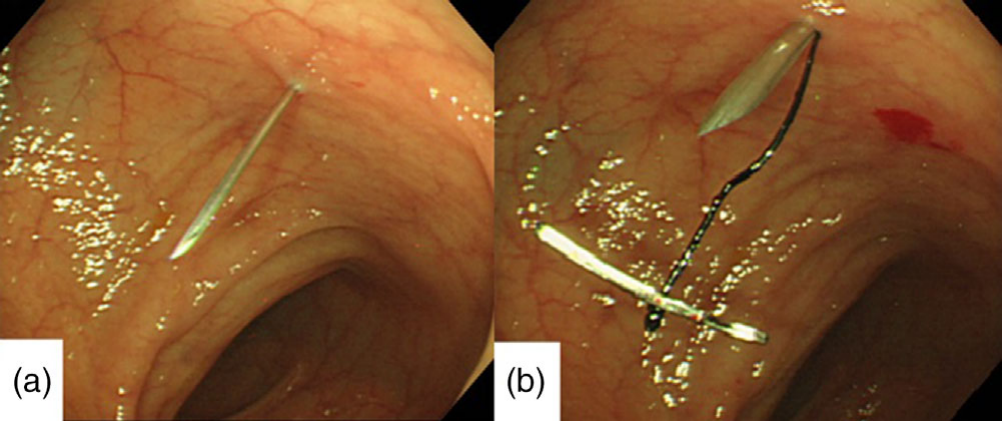
(a) Test puncture with a 23‐gauge needle did not cause perforation. (b) The T‐bar was implanted twice in the same area at different angles

**FIGURE 3:**
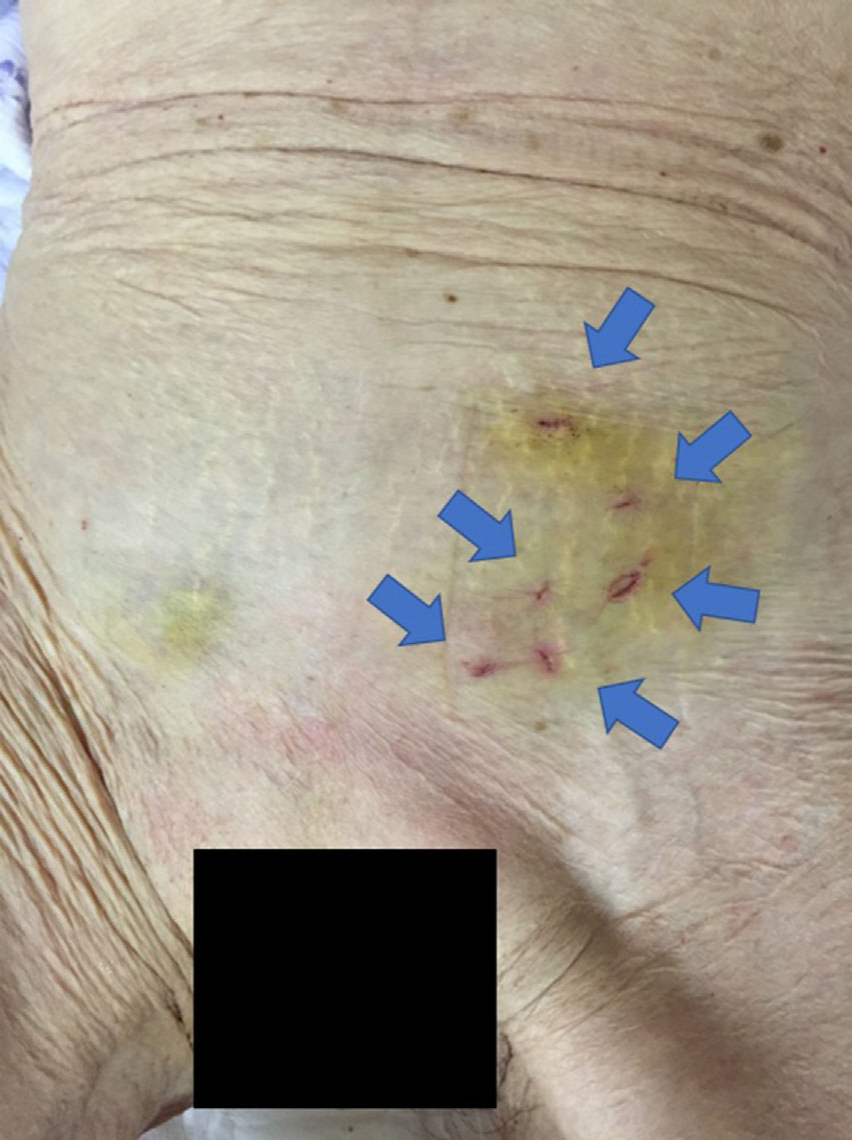
Colonoscopy‐assisted percutaneous sigmoidopexy is performed without laparotomy. The sigmoid colon is anchored to the abdominal wall at six sites (blue arrows)

1. A day before the surgery, bowel cleansing was performed using laxatives (50 g of magnesium citrate dissolved in 200 ml of water).
2. A colonoscope was inserted transanally under fluoroscopic guidance, and the prolapsed sigmoid colon was reduced.
3. The endoscope was then pushed into the anterior wall of the proximal rectum to straighten the intussuscepted sigmoid colon and approximate the puncture site.
4. As previously reported,^1^ a transmitted illumination test, a puncture test with a 23‐gauge needle, and an abdominal wall finger push test were performed at the designated fixation site; these tests ruled out the involvement of other organs, such as the small intestine, at this site. In the transmitted illumination test, the abdominal wall was observed for the transmission of light emitted from the endoscope's tip to confirm that it did not diffuse. The abdominal wall finger push test was performed to confirm that a finger‐shaped compressed image appeared on the endoscope when pressure was applied to the abdominal wall with a finger.
5. The fixation site was anesthetized with 1% xylocaine; using a scalpel, a 2 mm skin incision was then made at the site.
6. A two‐shot anchor (Olympus, Tokyo, Japan) was inserted into the lumen of the sigmoid colon (Figure 2a)^1^; one of the two nylon threads of the anchor, with a metal bar at its tip (T‐bar), was detached and pulled towards the body surface (Figure 2b).
7. The two‐shot anchor was similarly used to puncture through the subcutaneous tissue several millimeters away. The anchor's two nylon threads were then ligated subcutaneously.
8. The sigmoid colon was subsequently anchored to the abdominal wall at six sites, approximately 3 cm near the anal side, using the same technique (Figure 3).

The total procedure time was 30 min; the fixation procedure lasted for 18 min, and the total blood loss was 2 g.

### Postoperative course

**FIGURE 4:**
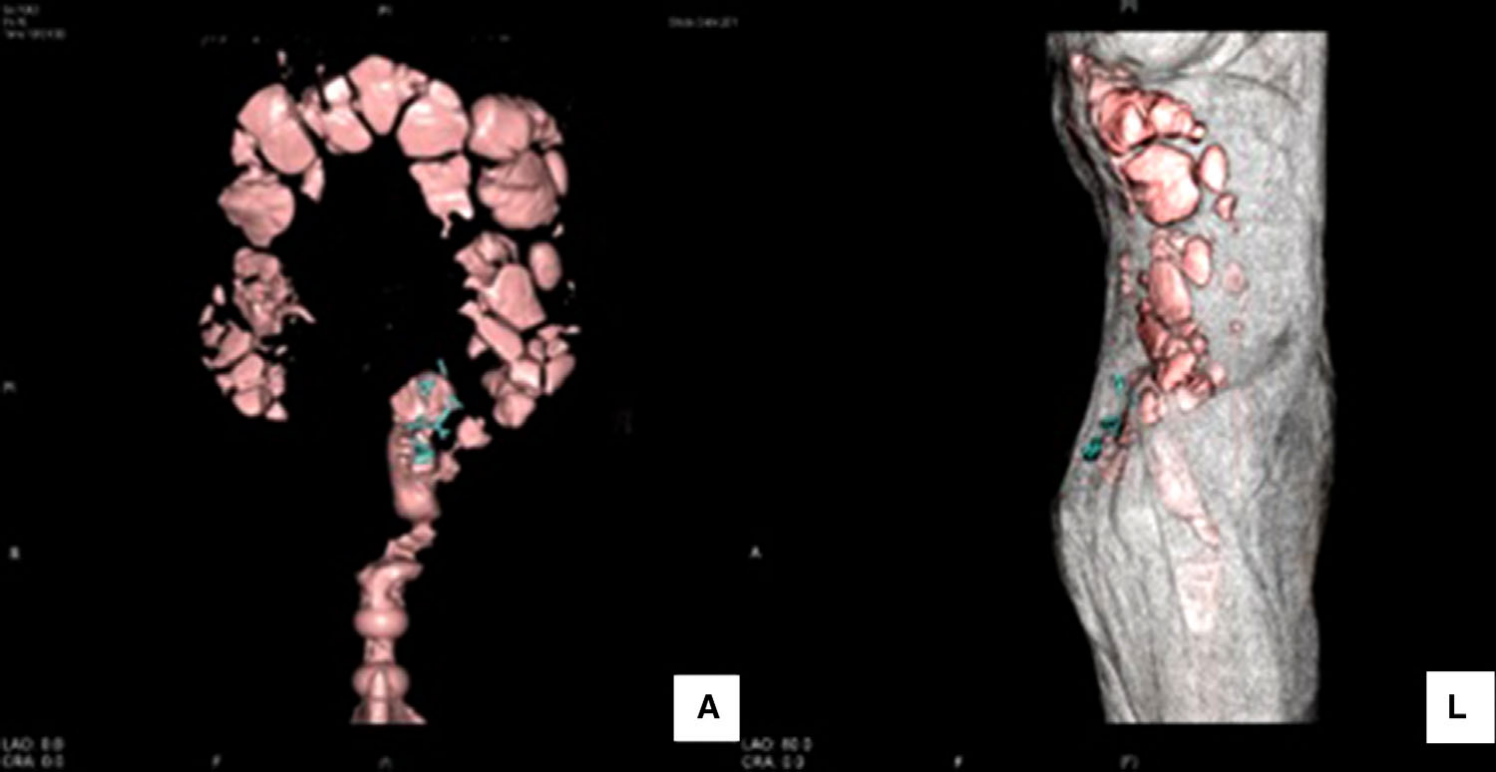
Computed tomography scan taken 5 years after the colonoscopy‐assisted percutaneous sigmoidopexy: T‐bars of the two‐shot anchor that fix the sigmoid colon and the rectum to the abdominal wall at a straightened angle. No recurrence was observed

The patient's bowel movements, fecal incontinence, and dysuria improved immediately a day after the surgery. She was able to walk and defecate on her own in the toilet, and she reported that she could feel the urge to defecate again. She did not experience constipation or abdominal straining during defecation. Her score on the Bristol scale was 3. Her status improved dramatically from being bedridden to walking unaided, and her Barthel Index improved to 55. No recurrence was observed during a 5‐year postoperative period (Figure 4).

## DISCUSSION

In this novel case, we performed CAPS to correct a complete rectal prolapse in a high‐risk older patient; this procedure was chosen due to its uncomplicated methodology, which did not require general anesthesia. No complications were observed.

Surgical treatments for a complete rectal prolapse include transperineal approaches (such as the Altemeier, Delorme, and Ganz–Miwa procedures) and a transabdominal approach (which necessitates sigmoid fixation to the anterior sacrum and sigmoid colon resection). Of these, transperineal approaches are often used in cases in which general anesthesia and other perioperative factors pose a high risk. However, perineal surgeries are associated with high 4‐year recurrence rates (14%–27%).^5^, ^6^, ^7^, ^8^, ^9^ For instance, the Gant–Miwa procedure (though simple) is associated with a high recurrence rate of approximately 30%.^6^ The Altemeier procedure reportedly decreases postoperative rectal compliance^9^; however, it is associated with a risk of suture failure because it necessitates intestinal anastomosis.

All aforementioned methods require approximately 100 min for completion.^9^ However, in the present case, CAPS was performed within 30 min; the procedure was shorter and less complicated as compared to the existing methods.

The patient experienced no complications or recurrence during the 5‐year postoperative course; she did not experience constipation, and her defecation control had improved.

Although this is a case report, we wish to discuss the indications, possibility of treatment failure, adverse effects, and postoperative precautions regarding CAPS on the basis of our previous experience with it during sigmoid volvulus management.

A history of surgery requires attention; due to concerns regarding small bowel injury, preoperative computed tomography scans should be carefully analyzed for possible small bowel adhesions to the abdominal wall at the fixation site. Intraoperative tests should be performed to rule out small bowel involvement.

Treatment failure refers to the occurrence of a prolapse immediately after endoscope removal. Because the procedure involves refluxing of the prolapsed bowel and its fixation in a fully stretched position, the likelihood of a recurrence after removal of the colonoscope seems low. However, the possibility of treatment failure during CAPS should be evaluated using a case series. While re‐fixation may be possible in such cases, its feasibility must be validated.

In terms of adverse events, subcutaneous emphysema was reported in one out of eight cases wherein CAPS was performed for sigmoid torsion. Furthermore, we have never encountered a case in which a metal rod was buried in the tissues following the use of the two‐shot anchor. However, large‐scale case series studies should be performed to assess the likelihood of other possible adverse events, such as peritonitis, small bowel injury, intra‐abdominal abscesses, bowel obstruction, and surgical wound infection.

Regarding postoperative precautions, surgeons must consider the possibility that a colonoscopy may be required during follow‐up; this is important because there are concerns regarding the feasibility of colonoscopy in patients after CAPS. While a colonoscopy was not performed in the present case, it was carefully and successfully executed following CAPS in one of our previous cases. However, its feasibility must be validated using a case series.

In conclusion, we report the first case of a complete rectal prolapse that was successfully treated using CAPS; future studies should use a case series to confirm the associated recurrence rates and complications.

## CONFLICT OF INTEREST

None.

## FUNDING INFORMATION

None.

## References

[1] Imakita T , Suzuki Y , Ohdaira H , Urashima M . Colonoscopy‐assisted percutaneous sigmoidopexy: A novel, simple, safe, and efficient treatment for inoperable sigmoid volvulus (with videos). Gastrointest Endosc 2019; 90: 514–20. 31077700 10.1016/j.gie.2019.04.246

[2] Lowry AC , Simmang CL , Boulos P *et al*. Consensus statement of definitions for anorectal physiology and rectal cancer: Report of the tripartite consensus conference on definitions for anorectal physiology and rectal cancer, Washington, D.C., May 1, 1999. Dis Colon Rectum 2001; 44: 915–9. 11496067 10.1007/BF02235475

[3] Wassef R , Rothenberger DA , Goldberg SM . Rectal prolapse. Curr Probl Surg 1986; 23: 397–451. 3522112 10.1016/0011-3840(86)90011-0

[4] Tsunoda A , Takahashi T , Matsuda S , Oka N , Kusanagi H . Midterm functional outcome after laparoscopic ventral rectopexy for external rectal prolapse. Asian J Endosc Surg 2020; 13: 25–32. 30920167 10.1111/ases.12701 PMC6972686

[5] D'Hoore A , Penninckx F . Laparoscopic ventral recto(colpo)pexy for rectal prolapse: Surgical technique and outcome for 109 patients. Surg Endosc 2006; 20: 1919–23. 17031741 10.1007/s00464-005-0485-y

[6] Pescatori M , Zbar AP . Tailored surgery for internal and external rectal prolapse: Functional results of 268 patients operated upon by a single surgeon over a 21‐year period*. Colorectal Dis 2009; 11: 410–9. 18637923 10.1111/j.1463-1318.2008.01626.x

[7] Pinheiro LV , Leal RF , Coy CS , Fagundes JJ , Martinez CA , Ayrizono Mde L . Long‐term outcome of perineal rectosigmoidectomy for rectal prolapse. Int J Surg 2016; 32: 78–82. 27345263 10.1016/j.ijsu.2016.06.040

[8] Altomare DF , Binda G , Ganio E *et al*. Long‐term outcome of Altemeier's procedure for rectal prolapse. Dis Colon Rectum 2009; 52: 698–703. 19404077 10.1007/DCR.0b013e31819ecffe

[9] Riansuwan W , Hull TL , Bast J , Hammel JP , Church JM . Comparison of perineal operations with abdominal operations for full‐thickness rectal prolapse. World J Surg 2010; 34: 1116–22. 20127331 10.1007/s00268-010-0429-0

